# Thermal-responsive ultrasound activatable *in situ* production of therapeutics for real-time imaging and targeted tumor therapy

**DOI:** 10.7150/thno.101714

**Published:** 2025-03-18

**Authors:** Meng Du, Fengyi Zeng, Ting Wang, Jinghui Fang, Yuhao Chen, Likang Hou, Bin Li, Qingrong Xia, Zhen Yuan, Fei Yan, Zhiyi Chen

**Affiliations:** 1 Key Laboratory of Medical Imaging Precision Theranostics and Radiation Protection, College of Hunan Province, The Affiliated Changsha Central Hospital, Hengyang Medical School, University of South China, Changsha, Hunan, China.; 2 Department of Medical Imaging, The Affiliated Changsha Central Hospital, Hengyang Medical School, University of South China, Changsha, Hunan, China.; 3 Institute of Medical Imaging, Hengyang Medical School, University of South China, Hengyang, Hunan, China.; 4 Medical Imaging Centre, The First Affiliated Hospital, Hengyang Medical School, University of South China, Hengyang, Hunan, China.; 5 Third Affiliated Hospital of Guangzhou Medical University, Guangzhou, Guangdong, China.; 6 Faculty of Health Sciences, Centre for Cognitive and Brain Sciences, University of Macau, Taipa, Macau SAR, China.; 7 CAS Key Laboratory of Quantitative Engineering Biology, Shenzhen Institute of Synthetic Biology, Shenzhen Institute of Advanced Technology, Chinese Academy of Sciences, Shenzhen, Guangdong, China.

**Keywords:** Bacteria-mediated tumor therapy, Ultrasound bioeffect, Imaging guided, Inducible gene expression, Immunogenic cell death

## Abstract

**Rationale:** Engineered bacteria with tumor-targeting capabilities and genetic modification potential have emerged as promising vectors for targeted tumor therapy through the effective production and release of therapeutic molecules. Controlled gene expression and real-time monitoring of therapeutic protein expression in tumors are of great significance for optimizing the therapeutic effective of engineered bacteria production as well as anti-tumor effects.

**Methods:** We constructed engineered *E. coli* MG1655 cells using recombinant thermal plasmid pBV220 carrying the optical imaging reporter gene miRFP720 and the therapeutic gene Cytolysin A, which could be controlled expressed synchronously by thermal effect produced by focused ultrasound. The concentrations of miRFP720 and Cytolysin A expressed by engineered bacteria after focused ultrasound irradiation were detected* in vitro* and *in vivo* with western blot analysis and enzyme-linked immunosorbent assay (ELISA) analysis. The immune cell infiltration rate and inflammation factors after treatment were detected by flow cytometry and ELISA analysis. The anti-tumor efficacy of engineered bacteria alone or combined with anti-Programmed cell death 1 ligand 1 (aPD-L1) immunotherapy were evaluated on subcutaneous tumor models and orthotopic glioblastoma models.

**Results:** Upon ultrasound-controlled activation, the expression level of therapeutic molecule Cytolysin A as well as the optical imaging probe miRFP720 were enhanced. Importantly, the expression level of Cytolysin A could be monitored *in vivo* non-invasively by the signal intensity produced by miRFP720, which provided guidance for optimized ultrasound-mediated therapeutic production *in situ*. Furthermore, with optimized ultrasound activation, Cytolysin A not only exerted potent anti-tumor effects but also induced immunogenic cell death, enhancing the therapeutic efficiency of aPD-L1 immunotherapy for deep site tumor treatment.

**Conclusion:** In this study, a real-time imaging guided ultrasound activatable tumor targeted therapy mode was established. Utilizing the thermal effect of focused ultrasound, Cytolysin A-miRFP720 engineered bacteria can concurrently express the therapeutic molecule Cytolysin A and the imaging probe miRFP720, which optimized the expression level of therapeutic production by engineered bacteria through imaging guided ultrasound irradiation mode, and realized enhanced the therapeutic efficiency of local tumor anti-tumor efficiency as well as aPD-L1 immunotherapy.

## Introduction

Bacteria-mediated tumor therapy (BMTT) has garnered substantial attention as a cutting-edge approach for delivering therapeutic molecules 1. The bacteria employed in BMTT have been recognized as unique biological sensors due to their distinctive physiological characteristics. Extensive research has shown that the inherent ability of bacteria to invade and colonize solid tumors is closely linked to the hypoxic nature of the tumor microenvironment. In contrast to passive delivery strategy, bacteria can penetrate tumors actively through self-propulsion 2. The presence of pathogen-associated molecular patterns after bacteria infiltration in tumor can also stimulate the immune microenvironment by recruiting and activating immune cells, thus enhancing specific immune recognition 3. Synergy with immunotherapy can also be achieved due to the tumor-targeting capability of BMTT. Bacterial nanohybrids and genetically engineered bacteria carrying therapeutic genes can exert immune-stimulating effects by inducing immunogenic cell death and promoting the production of proinflammatory cytokines 4. These unique capabilities of bacteria make them well-suited as precise sensors that respond to the tumor microenvironment, enabling control over the accumulation of antitumor payloads at tumor sites and the timing of drug delivery.

With advancements in synthetic biology, therapeutic genes can be engineered with smart genetic circuits controlled by inducible promoters, allowing the local production of antitumor molecules within tumors and enhancing the therapeutic efficacy and safety of BMTT. Various inducible promoter systems, such as those responsive to chemical 5 and physical inducers 6, have been developed to control gene expression and reduce systemic toxicity. Chemical inducers 7, 8 are commonly used to regulate gene expression in engineered bacteria; however, they cannot be targeted to specific sites. Meanwhile, light-induced approaches offer high spatiotemporal accuracy 9 but are limited by the poor penetration of light into solid tissues 10. Radiation-inducible promoters can achieve deep tissue penetration 11, but radiation poses a high risk of damaging normal cells and engineered microbial cells 12.

Temperature-based control elements can enable deep spatiotemporal control using noninvasive methods such as ultrasound. Ultrasound has been applied as a safe tool for generating heat to induce gene expression, leveraging its cavitation and thermal effects through the penetration of sound waves in biological tissues. In our previous study, ultrasound-responsive bacteria (URB) engineered with the thermal-inducible plasmid PBV220 were developed 13. Under focused ultrasound irradiation, URB could express proteins of interest in the tumor area and exert potent anti-tumor effects *in vitro* and *in vivo*, providing a novel inducible gene expression mode with the advantages of deep tissue penetration and good spatiotemporal controllability. Therefore, with ultrasound irradiation, therapeutic molecules can be produced in tumor areas with high efficiency. However, at different stages of solid tumor development, the physicochemical properties and hypoxia levels of the tumor microenvironment vary greatly. The heterogeneity of solid tumors also leads to differences in the number of engineered bacteria aggregating in the tumor area and the expression level of therapeutic molecules after induction, ultimately reduces the therapeutic efficiency of engineered bacteria. To optimize the therapeutic efficiency of BMTT, it is crucial to monitor the *in vivo* distribution of engineered bacteria and the real-time expression level of therapeutic molecules after external induction.

Advancements in biomedical engineering and medical imaging have highlighted the potential of imaging reporter genes in improving the *in vivo* distribution of engineered bacteria and monitoring therapeutic protein expression in real-time 14, 15. Imaging reporter genes were widely recognized for their predominant use in monitoring target gene expression levels, dynamically observing molecular interactions between cells and proteins, and aiding *in vivo* cell tracking. In the context of BMTT, the imaging signal of engineered bacteria using reporter genes has been shown to aid in evaluating bacterial proliferation rates and detecting tumor metastases 15. Moreover, when controlled by the same promoter, the reporter gene demonstrates the potential to reflect the expression level of the therapeutic gene when co-expressed.

**Figure 1:**
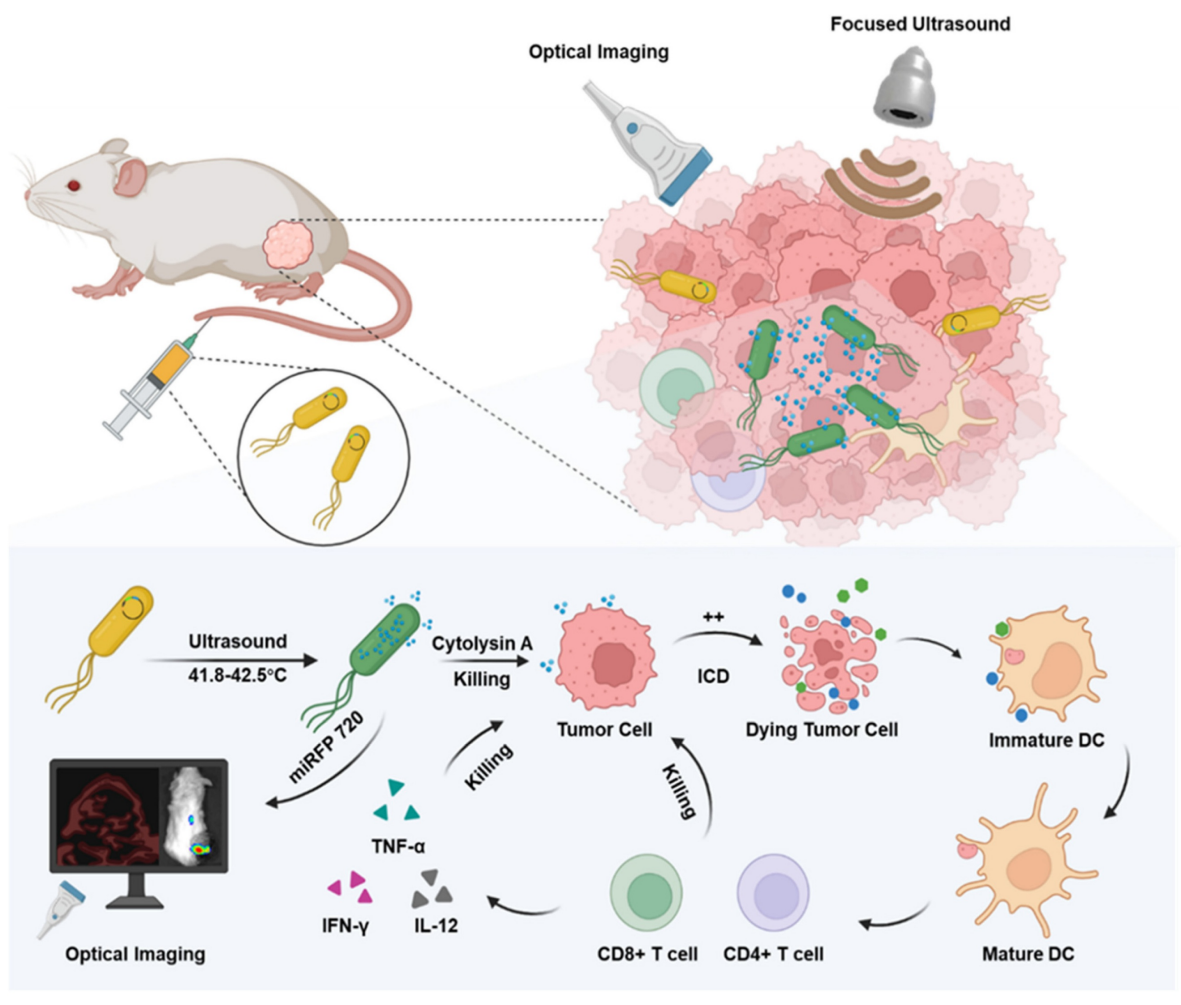
Schematic depiction of engineered bacteria-mediated ultrasound-controlled visualization of gene expression for tumor immunotherapy. The tumor-targeting engineered bacteria (Cytolysin A-miRFP720 engineered bacteria) were administered to tumor-bearing mice, and the expression of the Cytolysin A-miRFP720 gene was induced by ultrasound irradiation. Importantly, by monitoring the imaging signal of the optical imaging gene miRFP720, the expression level of the therapeutic protein Cytolysin A can be reflected, providing real-time imaging guidance for ultrasound irradiation to optimize the expression of the Cytolysin A gene in the ultrasound-responsive gene circuit. The synthesis of Cytolysin A heightens tumor antigen exposure, fostering DCs maturation, triggering immunogenic cell death, stimulating CD4+ and CD8a+ T cells, and stimulating cytokines like interferon-γ (IFN-γ), tumor necrosis factor-α (TNF-α), and interleukin-12 (IL-12). Consequently, this significantly bolsters the anti-tumor response.

In this study, engineered *Escherichia coli* MG1655 cells were constructed with the reconstructed thermal sensitive plasmid pBV220 carrying the optical imaging gene miRFP720 and the therapeutic gene Cytolysin A. Both genes were integrated into the multi-cloning site, regulated by the same lambda pL/pR promoter but two different ribosomes (RBS), which meant when the promoter was activated, both Cytolysin A and miRFP720 were produced synchronously but respectively. To realize the goal of therapeutic molecule secretion and achieve ideal anti-tumor effect, a classical secreted peptide gene was designed at the beginning of sequence of Cytolysin A. To detect the expression of Cytolysin A quantitatively, a classical 6×His-tag gene sequence was designed at the end of the sequence of Cytolysin A. As for the working principle of plasmid, pBV220 was constructed with a lambda pL/pR temperature-regulated progenitor and a heat-sensitive cI857 suppressor, which inhibits transcription from the promoter or the tandem promoter at relatively low temperatures (28-30°C). With heat stimulation changing the environment temperature to 42-45°C, such as thermal effect of focused ultrasound, cI857 suppressor was rapidly inactivated, which cause expression of Cytolysin A and miRFP720. The expression level of miRFP720 could be detected using optical imaging systems and reflected the location of bacteria as well as the relative expression levels of Cytolysin A, which can realize therapeutic gene visual monitoring *in vivo* and guide the therapeutic mode precisely. Cytolysin A could not only exert anti-tumor effect directly, but also induce immunogenic cell death, promoted DCs maturation, and activated CD4+ and CD8a+ T cells, enhancing aPD-L1-based immunotherapy (Figure 1).

## Materials and Methods

### Construction of Cytolysin A-miRFP720 engineered bacteria

The pBV220 plasmid was purchased from Wuhan Miaoling Bioscience Co. Ltd. The Cytolysin A-miRFP720 gene, with the OmpA gene sequence at the beginning of Cytolysin A, 6×His-tag gene at the end of Cytolysin A gene, and the RBS between Cytolysin A and miRFP720 was synthesized by Sangon Biotech Co., Ltd. (Shanghai, China). Using EcoRI and BamHI as restriction enzymes, the synthesized sequences were inserted into the pBV220 plasmid. The sequences of recombined plasmid were confirmed by enzyme digest experiment, which applied agarose gel electrophoresis to analyze the band distribution with or without enzyme digesting. The plasmids were transferred into *E. coli* MG1655 competent cell (Angyu Biotech Co., Ltd, Shanghai, China) through chemical transformation to construct engineered bacteria (Cytolysin A-miRFP720 engineered bacteria). The genetically modified bacterial strains were subsequently grown on LB agar with 100 μg/mL ampicillin at 30 °C for one night. Positive colonies were then cultured in LB liquid medium containing 100 μg/mL ampicillin at 30 °C with shaking at 200 RPM.

### Establishment of the methods of ultrasound-regulated gene expression in engineered bacteria

The Cytolysin A-miRFP720 engineered bacteria were grown overnight at 30 °C with shaking at 200 RPM and diluted to 10^8^ CFU/mL. The focal length and radius of the focal point of focused ultrasound were calculated by virtual sound field scanning. The bacterial solution was irradiated using focused ultrasound under a series of acoustic pressures, including 2.82 MPa, 3.17 MPa, 3.52 MPa, 3.87 MPa, and 4.22 MPa. The temperature change of the bacterial solution was measured using an infrared thermal imager. After confirming the optimal acoustic pressure condition, the bacterial solution was irradiated for various durations (0, 10, 20, 30 min), and the temperature was controlled between 41.8 °C and 42.5 °C using an ON/OFF switch. The optical density at 600 nm and the counts of bacteria clones on LB agar was recorded for evaluating the viability of Cytolysin A-miRFP720 engineered bacteria with various ultrasound irradiation mode. The optical imaging signal of miRFP720 was recorded through IVIS Spectrum system from PerkinElmer, with excitation/emission wavelengths of 645/720 nm. The bacteria culture samples before ultrasound irradiation and after ultrasound irradiation (30 min) was collected for transmission electric microscope observation.

### Cell culture

4T1 tumor cells were purchased from the BeNa Culture Collection. Cells were grown in high-glucose medium containing 10% fetal bovine serum and 1% penicillin-streptomycin. Murine bone marrow-derived DCs were isolated from 6-week-old female BALB/c mice. In brief, bone marrow from mice was collected by flushing the femur and tibia with PBS, followed by lysis of red blood cells. The remaining cells were rinsed twice with PBS and then placed in complete RPMI 1640 medium supplemented with recombinant murine granulocyte-macrophage colony-stimulating factor (GM-CSF) at a concentration of 20 ng/mL (MedChemExpress, USA) for six days to generate immature DCs. On day seven, immature DCs were collected.

### Tumor-bearing mouse models

All animal experiments described in this study were performed in accordance with institutional guidelines and approved by the Institutional Animal Care and Use Committee (IACUC) of the Animal Experiment Center of Shenzhen Institutes of Advanced Technology, Chinese Academy of Science (SIAT-IACUC-190620-YGS-YF-A0766). All animal experiments were complied with the ARRIVE guidelines. For subcutaneous tumor implantation, 5-6-week-old female BALB/c mice were implanted in the mid-right flank with 5×10^6^ 4T1 tumor cells. Tumor-bearing mice were randomly allocated into 4 groups once their tumor volumes reached 100 mm^3^.

For *in situ* glioblastoma multiforme (GBM) tumor implantation, 5×10^5^ GL261-Luc cells in 5 μL of DMEM were injected into the right cerebral hemisphere of anesthetized mice. The exact position of injection was 1 mm lateral to the bregma at a depth of 5 mm. Bioluminescence imaging was conducted ten days' post-injection to confirm the successful creation of the GBM model.

### Optimization of ultrasound-controlled gene expression through imaging guidance

The culture methods of Cytolysin A-miRFP720 engineered bacteria was described above. With various temperature controlled by ultrasound irradiation for 30 min, bacterial protein of different group was collected. Protein Electrophoresis with 12% sodium dodecyl sulfate-polyacrylamide gel and gel stained with Coomassie brilliant blue dye was performed to prove the expression of Cytolysin A-miRFP720 gene after ultrasound irradiation. In order to analysis the relationship between imaging signal of miRFP720 and concentration of Cytolysin A *in vitro* and *in vivo*, immunoblot analysis with a polyclonal anti-His-tag and His-tag enzyme-linked immunosorbent assay (ELISA) analysis was performed. In order to prove the advantage of imaging guided optimized ultrasound-controlled gene expression mode, tumor bearing mice were divided into US once group, which received regular ultrasound irradiation mode, and US Twice group, whose timing of ultrasound irradiation was determined by the dynamic change of fluorescence signal of miRFP720.

### Cytolysin A-mediated cell cytotoxicity

Cell Counting Kit-8 (CCK-8) assay was applied to evaluate the viability of 4T1 tumor cells. The supernatant of Cytolysin A-miRFP720 engineered bacteria was passed through a 0.22 μm filter to remove bacteria after ultrasound induction. In order to detect the expression level of Cytolysin A of supernatant of engineered bacteria, the supernatant was concentrated 30-fold using trichloroacetic acid and acetone. ELISA analysis was used to quantify the concentration of His-tagged Cytolysin A protein in the supernatant. 4T1 tumor cells were seeded into 96-well plates (1×10^5^ cells per well) for 24 h at 37 °C and 5% CO_2_, followed by a 12 h treatment with supernatants of Cytolysin A-miRFP720 engineered bacteria at various concentrations. After treatment, cells were washed three times with PBS and incubated with fresh medium containing 10% CCK-8 for 4 h. The optical density at 450 nm was measured with microplate reader. The relative cell viability was measured according to standard protocols. Briefly, staining working solution was composed of 5 μL of Calcein-AM solution (2 mM), 15 μL of propidium iodide solution (1.5 mM), and 5 mL of 1× assay buffer. 100 μL of the prepared working solution was added to each well and incubated for 15 min. Fluorescence microscope was applied to observe the signal of fluorescence in wells.

### Induction of immunogenic cell death by Cytolysin A produced by engineered bacteria after ultrasound induction

The exposure to DAMPs, such as HMGB1 and CRT, from tumor cells after different treatments *in vitro* was measured. 4T1 tumor cells were seeded into 96-well plates (8×10^3^ cells per well) and incubated in 100 μL DMEM medium containing 10% FBS for 12 h. The bacterial culture supernatant with corresponding concentrations of His-tagged Cytolysin A protein (2-6 ng/mL) was added to the tumor cells seeded in 96-well and incubated for 48 h. The exposure of CRT was measured using immunofluorescence detection with a confocal laser scanning microscope after 12 h (Calreticulin Rabbit Monoclonal Antibody, Cat#AF1666, 1:500, Beyotime, China). A semi-quantitative analysis was performed using ImageJ software. After incubating the cells for 48 hours, the supernatant from the cell culture was collected and utilized for HMGB1 detection through ELISA analysis following the manufacturer's instructions. Immature DC cells incubated with 4T1 tumor cells which had been previously treated with PBS, 2 ng/mL Cytolysin A protein, or 6 ng/mL His-tagged Cytolysin A protein 24 h prior. After a 48-h co-incubation, DCs were stained with the indicated antibodies: APC-CD86 (Cat#105011, BioLegend, USA) and CD11c (Cat#12-5321-81, ThermoFisher, USA). The cells were then analyzed by flow cytometry.

For *in vivo* experiments, CRT and HMGB1 exposure levels were analyzed using immunofluorescence and confocal laser scanning microscopy. Flow cytometry was used to analyze intratumoral immune cells. Tumors were collected seven days after treatment and cut into small pieces. The tissue was then digested using collagenase V (2 mg/mL, YEASEN) in Hank's Balanced Salt Solution (HBSS) for 4 h at 37 °C. Cells were filtered through a 70 μm cell strainer to obtain a single-cell suspension and washed with fluorescence-activated cell sorting (FACS) buffer. The single-cell suspension was pre-blocked with antibodies for 30 min at 4 °C. Then, the cells were stained with surface antibodies. Three kinds of immune cells were detected in these experiments, including mature dendritic cells (mDCs), CD4+ T cells and CD8a+ T cells. The data were analyzed using FlowJo software. As for the gating strategies of different immune cells during analyzing with FlowJo, FSC and SSC were applied for selecting the represented immune cell group, which provided the basic cell group for further analysis. DCs were stained with anti CD11c FITC (Cat# 117305, BioLegend, USA) and then CD86 PE (Cat# 105105, BioLegend, USA) and CD80 APC (Cat# 104713, BioLegend, USA) was used for analyzing mDCs. T cells were stained with anti CD3 FITC (Cat# 100203, BioLegend, USA), and CD4 APC (Cat# 100412, BioLegend, USA) and CD8a PE-Cy7 (Cat# 100721, BioLegend, USA) was respectively used for analyzing CD4+ T cells and CD8a+ T cells. The intratumoral levels of IFN-γ, TNF-α, and IL-12 after the treatment were detected with ELISA kits according to the manufacturer's instructions.

### *In vivo* biodistribution of Cytolysin A-miRFP720 engineered bacteria

IVIS Spectrum was applied to evaluate the biodistribution of the signal of miRFP720 after administration of bacteria or bacterial lysate. The optical characteristics of miRFP720 allowed us to use miRFP720 as a monitoring probe for the engineered bacteria after ultrasound irradiation. Ultrasound radiation was used to heat the bacterial culture to 42 °C for 2 h. After ultrasound irradiation, bacteria were cultured for another 4 h at 30 °C with shaking at 200 RPM. The bacterial solution was split into two equal parts, each containing 10^8^ CFU/mL of bacteria. One aliquot was used for intravenous administration, and the other aliquot was used to extract bacterial lysate by ultrasonic lysis for injection into tumor-bearing mice. The distribution of Cytolysin A-miRFP720 engineered bacteria was monitored using fluorescence imaging at 675/720 nm within two days after administration. Mice were sacrificed and tumor tissue as well as major organ, including heart, liver, spleen, lung and were removed for fluorescence imaging. Furthermore, on the first, second, and seventh day after administration, organs were homogenized, diluted, and cultured on LB agar. After culture overnight, the number of colony-forming units (CFUs) was counted.

### *In vivo* photoacoustic imaging of Cytolysin A-miRFP720 engineered bacteria

To observe the biodistribution of CytolysinA- miRFP720 engineered bacteria with photoacoustic imaging *in vivo*, 10^8^ CFU/mL of Cytolysin A-miRFP720 engineered bacteria treated as mentioned above were administered to tumor-bearing mice via tail vein injection. Photoacoustic imaging was used to observe the photoacoustic signal of the tumor margin and tumor core.

### *In vivo* antitumor immunity of Cytolysin A-miRFP720 engineered bacteria

In subcutaneous tumor experiments, tumor-bearing mice were segregated into four groups: PBS, ultrasound irradiation combined with administration of wild-type MG1655 (US+WT-MG1655), administration of Cytolysin A-miRFP720 engineered bacteria alone (Cytolysin A-miRFP720), and ultrasound irradiation combined with administration of Cytolysin A-miRFP720 engineered bacteria (US + Cytolysin A-miRFP720). All mice were treated with tail vein injection. WT-MG1655 and Cytolysin A-miRFP720 engineered bacteria (10^8^ CFU) were administered on days 1 and 7. At two and four days after bacteria administration, ultrasound irradiation (3.52 MPa, 30 min, 42 °C) of the local tumor site was performed (Figure 6A). The volumes of tumor and body weights of tumor bearing mice were detected every two days. For biosafety and immunogenicity evaluation, the organs and blood of mice of Control and US + Cytolysin A-miRFP720 engineered bacteria group were collected for histological examinations and blood chemistry analysis.

For orthotopic glioblastoma treatment experiments, tumor-bearing mice were divided into six groups: control, Cytolysin A-miRFP720 engineered bacteria, aPD-L1, US + Cytolysin A-miRFP720 engineered bacteria, US + aPD-L1, and US + Cytolysin A-miRFP720 engineered bacteria + aPD-L1. 110 μL of PBS, aPD-L1 (100 μg/mL), or engineered bacteria (1×10^5^ CFU/mL) was intravenously injected. After 48 h, ultrasound radiation at 3.52 MPa was used to irradiate the engineered bacteria in the US + Cytolysin A-miRFP720 engineered bacteria, US + aPD-L1, and US + Cytolysin A-miRFP720 engineered bacteria + aPD-L1 groups for 30 min. At the end of monitoring on day 15, mice were sacrificed, and the main organs (heart, liver, spleen, lung, kidney, and tumor) were harvested (Figure 7A). Organs were fixed in 4% paraformaldehyde, embedded in paraffin, cut into 5 μm slices, and stained with hematoxylin and eosin. Slides were then examined under a light microscope. Apoptosis and angiogenesis in tumor tissues were also studied using immunofluorescence staining for TUNEL and CD31, respectively.

## Results and Discussion

### Development of ultrasound-regulated gene expression pathways in Cytolysin A-miRFP720 engineered bacteria

**Figure 2:**
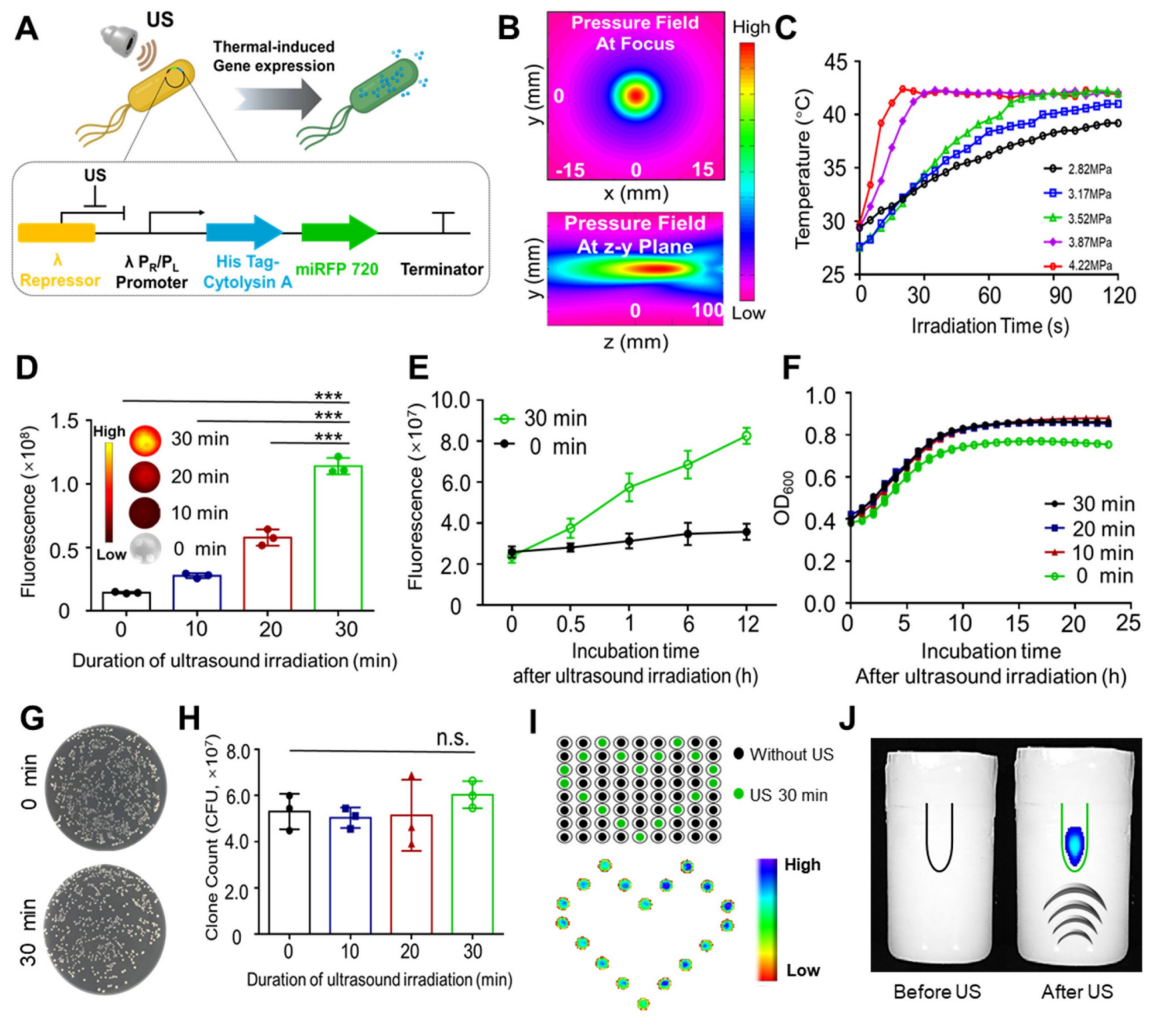
Demonstration of the mechanism for ultrasound-regulated gene expression in Cytolysin A-miRFP720 engineered bacteria. (A) Diagram of the process of ultrasound-controlled gene expression in Cytolysin A-miRFP720 engineered bacteria and the key elements of recombined plasmid. (B) The acoustic field of the focused ultrasound irradiation setup. (C) The temperature changes of engineered bacteria under ultrasound irradiation at a series of acoustic pressures. (D) Quantification of fluorescence signals of engineered bacteria (5×10^8^ CFU/mL) solution after ultrasound irradiation for various durations. Data are representative of three independent experiments. (E) Measurement of the continuous fluorescence signal within 12 h after focused ultrasound irradiation. Optical density measurements at 600 nm of engineered bacteria culture after ultrasound irradiation in a time series (F) and colony counting on LB agar (G). (H) Counts of engineered bacteria colonies following ultrasound exposure for different durations. (I) Fluorescence signal emitted by engineered bacteria in 96-well plates with and without ultrasound exposure. (J) The fluorescence signal of engineered bacteria in EP tube buried in agarose phatom before and after ultrasound irradiation. Data are presented as mean ± SD, and one-way ANOVA with Tukey's test was performed on GraphPad Prism. (^***^p<0.001, n.s. not significant).

A previous study reported a highly spatiotemporally controllable gene expression strategy that triggered bacterial *in situ* interferon-γ (IFN-γ) expression by ultrasound, showing promising applications in tumor therapy 13. It is important to monitor and maintain high level expression of therapeutic production from engineered bacteria *in vivo* through optimized induction method. Therefore, in this study, the aim was to develop novel ultrasound activated engineered bacteria capable of reflecting the expression level of therapeutic genes through imaging signals. To achieve this goal, engineered bacteria were developed with the recombinant pBV220 plasmid carrying the genes encoding the imaging probe miRFP720 and the therapeutic molecule Cytolysin A (Cytolysin A-miRFP720), controlled by the same heat-sensitive pR/pL promoter but different RBS (Figure 2A). The sequence of pBV220-Cytolysin A-miRFP720 was confirmed by agarose gel electrophoresis of plasmid and enzyme digested product (Figure S1). The thermal effect of focused ultrasound, with the focal length at 22 cm and focal point radius at 1 mm (Figure 2B), was applied to trigger the expression of miRFP720 and Cytolysin A. To optimized the ultrasound irradiation parameter, a series of acoustic pressures ranging from 2.82 MPa to 4.22 MPa were tested. As Figure 2C shown, under an acoustic pressure of 3.52 MPa, the temperature of bacterial culture irradiated by ultrasound rose steadily and was maintained at 42 °C, which was selected as the optimized parameter for subsequent experiments.

Based on the optical characteristics of miRFP720 16, fluorescence imaging was used to evaluate the gene expression level after ultrasound irradiation. With a 30-min ultrasound irradiation duration, the fluorescence signal increased approximately 6-fold and continued to increase during 12 h after ultrasound irradiation, compared to the control group (Figure 2D, 2E). These results proved the feasibility of ultrasound-controlled gene expression in engineered bacteria.

The influence of various ultrasound irradiation durations (0-30 min) on the viability of engineered bacteria was further examined. By evaluating the changes of the integrity of the bacteria, optical density at 600 nm of bacterial cultures and counting the colonies of Cytolysin A-miRFP720 engineered bacteria, it was proved that the appearance as well as the growth profile of Cytolysin A-miRFP720 engineered bacteria after ultrasound irradiation was slightly higher compared to the control group (Figure 2F). The number of colonies formed from Cytolysin A-miRFP720 engineered bacteria culture after ultrasound irradiation was similar to that of non-irradiated bacteria, indicating that ultrasound irradiation for up to 30 min did not affect the viability of Cytolysin A-miRFP720 engineered bacteria (Figure 2G-H). What's more, the results of transmission electricity microscope showed that compared to control group that received none ultrasound irradiation, the outlook and integrity of bacteria has not changed, even received ultrasound irradiation for 30 min (Figure S2).

Ultrasound as a stimulation method possesses the advantage of good specificity and deep penetration ability, providing a suitable mode of gene controlled expression for BMTT. To prove this, two interesting experiments were performed. First, we added Cytolysin A-miRFP720-engineered bacterial culture to each sample well of the 96-well plate, and performed ultrasound irradiation according to the planned route. As the fluorescent imaging showed, the irradiated cultures in wells formed a romantic pattern due to the specific trajectory of the ultrasound (Figure 2I). In another experiment, an EP tube with Cytolysin A-miRFP720 engineered bacteria culture was buried in an agarose phantom, and ultrasound transducer was placed under the phantom. With ultrasound irradiation from a remote site, the fluorescence signal could be induced, attributed to the deep penetration capability of focused ultrasound (Figure 2J).

**Figure 3:**
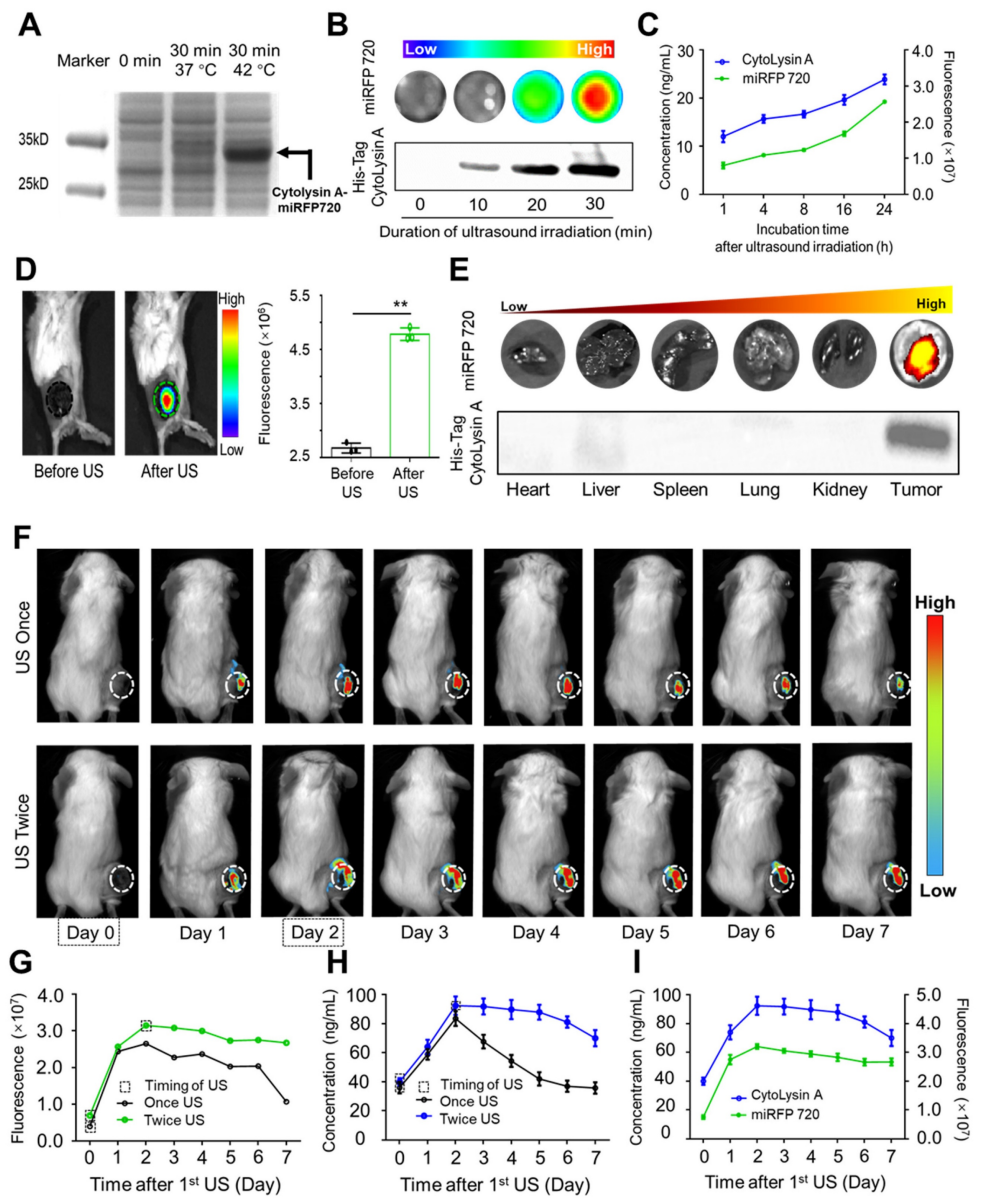
Imaging visualization of ultrasound-controlled gene expression in Cytolysin A-miRFP720 engineered bacteria. (A) Image of Band distribution of Cytolysin A-miRFP720 protein produced by engineered bacteria after ultrasound induction on SDS-polyacrylamide gel. (B) Optical imaging signal and western blotting analysis of engineered bacteria culture with various ultrasound irradiation duration *in vitro* (0, 10, 20, 30 min). (C) The correlation curve between the fluorescence intensity of miRFP720 and the concentration of His-tagged Cytolysin A protein within 24 h after ultrasound irradiation *in vitro*. (D) Fluorescence image of tumor-bearing mice received engineered bacteria administration, and quantitative analysis of fluorescent signal of tumor area, before and after ultrasound irradiation. (E) Fluorescence images and western blotting analysis of major organs and tumor tissues from tumor-bearing mice received engineered bacteria administration and ultrasound irradiation. (F) Fluorescence signal of miRFP720 in tumor tissues with different ultrasound irradiation mode at various time points (0, 1, 2, 3, 4, 5, 6, 7 days after 1^st^ ultrasound irradiation). (G) Quantitative analysis of the fluorescence intensity of miRFP720 *in vivo* and (H) the expression level of His-tagged Cytolysin A protein *ex vivo* from tumor bearing mice with different ultrasound irradiation mode. (I) Correlation between the expression of His-tagged Cytolysin A protein and the fluorescence signal of miRFP720 *in vivo* from (G, H). Data are presented as mean ± SD, and Student's t-test was performed on GraphPad Prism. (^**^p<0.01).

As shown by SDS-polyacrylamide gel electrophoresis, the pR/pL promoters of the recombinant pBV220 plasmid could be activated by focused ultrasound to simultaneously release miRFP720 (34.7 kD) and Cytolysin A (33.5 kD) (Figure 3A). In order to determine the expression level of the targeted gene after induction, a 6 × His tag was attached to the C-terminus of Cytolysin A. Based on this principle, the correlation between the fluorescence signal of miRFP720 and the band thickness of His-tagged Cytolysin A protein on a protein gel was analyzed. The fluorescence signal of miRFP720 increased with the ultrasound irradiation duration, while the expression level of His-tagged Cytolysin A protein also increased with the same trend (Figure 3B). According to these results, it was confirmed that the fluorescence signal of miRFP720 has positive correlation with the expression rate of His-tagged Cytolysin A protein. Furthermore, the fluorescence signal of miRFP720 and the concentration of Cytolysin A were quantified using His-tag ELISA analysis. It was found that both the fluorescence signal and the concentration of His-tagged molecules increased synchronously within 24 h after ultrasound irradiation, providing verification for the application of imaging signals in quantitatively monitoring therapeutic gene expression at the molecular level (Figure 3C).

*In vitro* experiments showed that the fluorescence signal of miRFP720 and the protein gel band thickness equally approximated the concentration of His-tagged Cytolysin A protein from engineered bacteria. *In vivo* experiments further revealed that the fluorescence signal of tumors could be enhanced after optimized ultrasound irradiation following intravenous administration of engineered bacteria (Figure 3D). Moreover, western blotting of isolated organs and tumor tissues demonstrated that His-tagged Cytolysin A protein was only expressed in the tumor, while other organs did not show evidence of Cytolysin A protein expression. The optical signal of isolated organs and tumor also showed the same trend with Cytolysin A (Figure 3E).

It is well known that* ex vitro* analysis of body fluids and tissues is a common method to determine protein expression levels. However, advancements in biomedical engineering technology, especially imaging technology, have made it possible to monitor protein expression *in vivo*. For example, optical imaging signal can be applied in distinguishing the different molecular compositions of atherosclerotic plaques *in vivo*. Considering the importance of monitoring therapeutic gene expression in engineered bacteria and the potential for continuous expression of Cytolysin A protein to enhance the antitumor effect, the necessity of imaging-guided ultrasound irradiation was aimed to be confirmed. After administration of Cytolysin A-miRFP720 engineered bacteria and the first ultrasound irradiation, the fluorescence signal of miRFP720 in the tumor area was used as a guide to determine the dynamic changes in Cytolysin A concentration *in vivo*. As depicted in Figure 3F, the fluorescence signal rose and peaked within the initial two days following ultrasound exposure. On day 2 after the first ultrasound irradiation, second ultrasound irradiation was performed and intended to maintain the expression level of Cytolysin A at tumor area. Compared to the single ultrasound group, tumor-bearing mice that received twice ultrasound irradiations exhibited a prolonged imaging signal peak (Figure 3G). As expected, the concentration of Cytolysin A in the tumor area that received twice ultrasound irradiations, as detected through tumor tissue homogenate and ELISA analysis, did not show an obvious decline compared to the single ultrasound group (Figure 3H) and exhibited a positive correlation with the fluorescence signal (Figure 3I). Based on this, with imaging signal guidance, it is possible to maintain therapeutic gene expression at a relatively high level, providing an optimal therapeutic mode for tumor treatment.

### *In vitro* antitumor efficacy of secreted Cytolysin A from engineered bacteria after ultrasound irradiation

**Figure 4:**
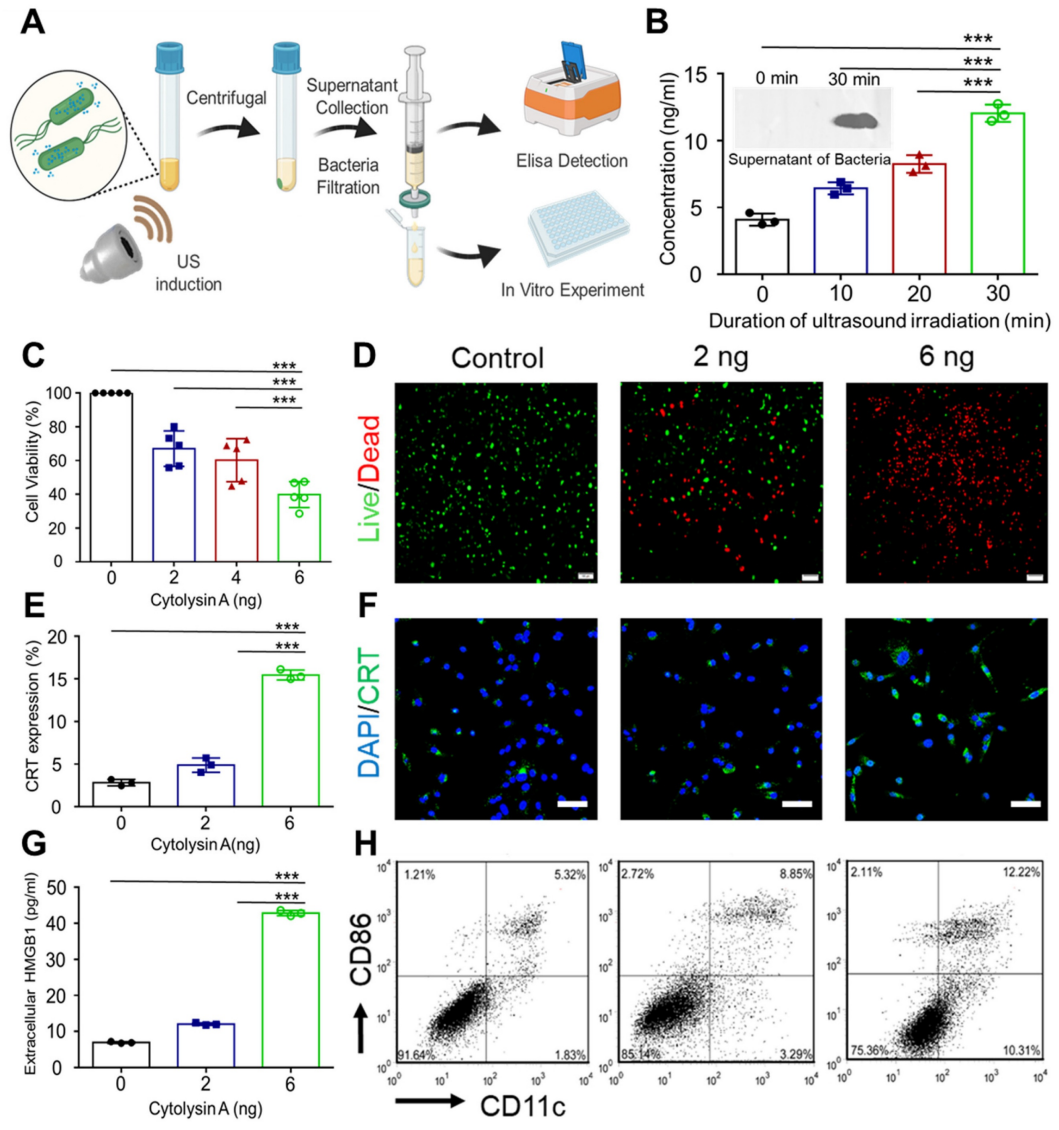
Assessment of *in vitro* antitumor efficacy and induction of immunogenic cell death by secreted Cytolysin A from the supernatant of engineered bacteria after ultrasound irradiation. (A) The process for collection and quantitative analysis of secreted Cytolysin A from engineered bacteria. (B) The concentration of His-tagged Cytolysin A protein was quantified after various ultrasound irradiation duration (0, 10, 20, 30 min) using His-tag ELISA analysis. Supernatants were also subjected to western blotting to visualize the His-tagged Cytolysin A protein band before and after ultrasound irradiation. (C) The viability of 4T1 tumor cells after incubation with different concentrations of Cytolysin A protein (0, 2, 6 ng/mL). (D) Fluorescence microscopy of 4T1 tumor cells stained with Calcein-AM (green) and propidium iodide (PI) (red) after incubation with different concentrations of Cytolysin A protein supernatants. (E-F) Fluorescence microscopy image and quantitative analysis of CRT (green) in 4T1 tumor cell (Blue) supernatants after incubation with different concentrations of secreted Cytolysin A protein. (G) Quantitative expression of high mobility group box 1 (HMGB1) in 4T1 tumor tumor cell supernatants concentrations of Cytolysin A protein after incubation with different concentrations of Cytolysin A protein. (H) The maturation rate of DCs after different treatment. Data are represented by three independent experiments. Data are presented as mean ± SD, and one-way ANOVA with Tukey's test was performed on GraphPad Prism. (^***^p<0.001).

Therapeutic molecules are crucial for the therapeutic efficiency of BMTT. Various therapeutic molecules produced by engineered bacteria have been proven effective for tumor treatment, including cytotoxic molecules, prodrug-converting enzymes, and immunomodulators 17. Cytolysin A is a bacterial toxin that can directly exert antitumor effects through pore formation 18. In this part, the antitumor efficacy of Cytolysin A produced by Cytolysin A-miRFP720 engineered bacteria after ultrasound irradiation was aimed to be evaluated *in vitro*. The OmpA signal sequence at the N-terminus of the Cytolysin A gene allowed for the successful release of Cytolysin A from Cytolysin A-miRFP720 engineered bacteria after ultrasound induction. The process of collection and analysis of secreted Cytolysin A from the supernatant of engineered bacteria was shown as Figure 4A. The concentration of secreted His-tagged Cytolysin A protein was first measured, with various irradiation duration of ultrasound performed on bacterial culture (0, 10, 20, 30 min). The concentration of His-tagged Cytolysin A protein in supernatants of the engineered bacteria was significantly enhanced with ultrasound irradiation, resulting in a 3-fold increase in protein production after 30 min of ultrasound irradiation compared to the non-irradiated group (Figure 4B). To evaluate the antitumor effect of His-tagged Cytolysin A protein secreted from engineered bacteria after ultrasound irradiation, CCK-8 assay analysis was utilized to examine the relative viability of 4T1 tumor cells treated with bacterial supernatants containing different concentrations of His-tagged Cytolysin A protein. The viability of 4T1 tumor cells consistently decreased as the concentrations of His-tagged Cytolysin A protein increased in cell culture (Figure 4C). Calcein AM/PI staining analysis showed that the proportion of dead cells increased with rising concentrations of His-tagged Cytolysin A protein (2 - 6 ng/mL) (Figure 4D). The above results proved that anti-tumor function of secreted Cytolysin A from engineered bacteria after ultrasound induction.

High mobility group box 1 (HMGB1) and Calreticulin (CRT), which are classic damage-associated molecular patterns (DAMPs), are prerequisite for DCs maturation to trigger immunogenic cell death-stimulated antitumor immunity 19. It has been reported that immunogenic cell death can enhance the sensitivity of immunotherapy, including PD-L1 blockade therapy 20. Therefore, the immune-regulating function of Cytolysin A, which has not been previously proven, was aimed to be verified. The expression and release of DAMPs in 4T1 tumor cells were examined after incubation with different concentrations of secreted Cytolysin A protein. It was proved that the expression of CRT after incubation with 6 ng His-tagged Cytolysin A protein was 5-fold higher than that of the control group (Figure 4E-F). What's more, the concentration of HMGB1 in the supernatant of 4T1 tumor cells increased with the concentration of secreted Cytolysin A protein (Figure 4G). With the stimulation of DAMPs, the maturation rate of DCs was also enhanced after incubation with secreted Cytolysin A, according to the Figure 4H.

**Figure 5:**
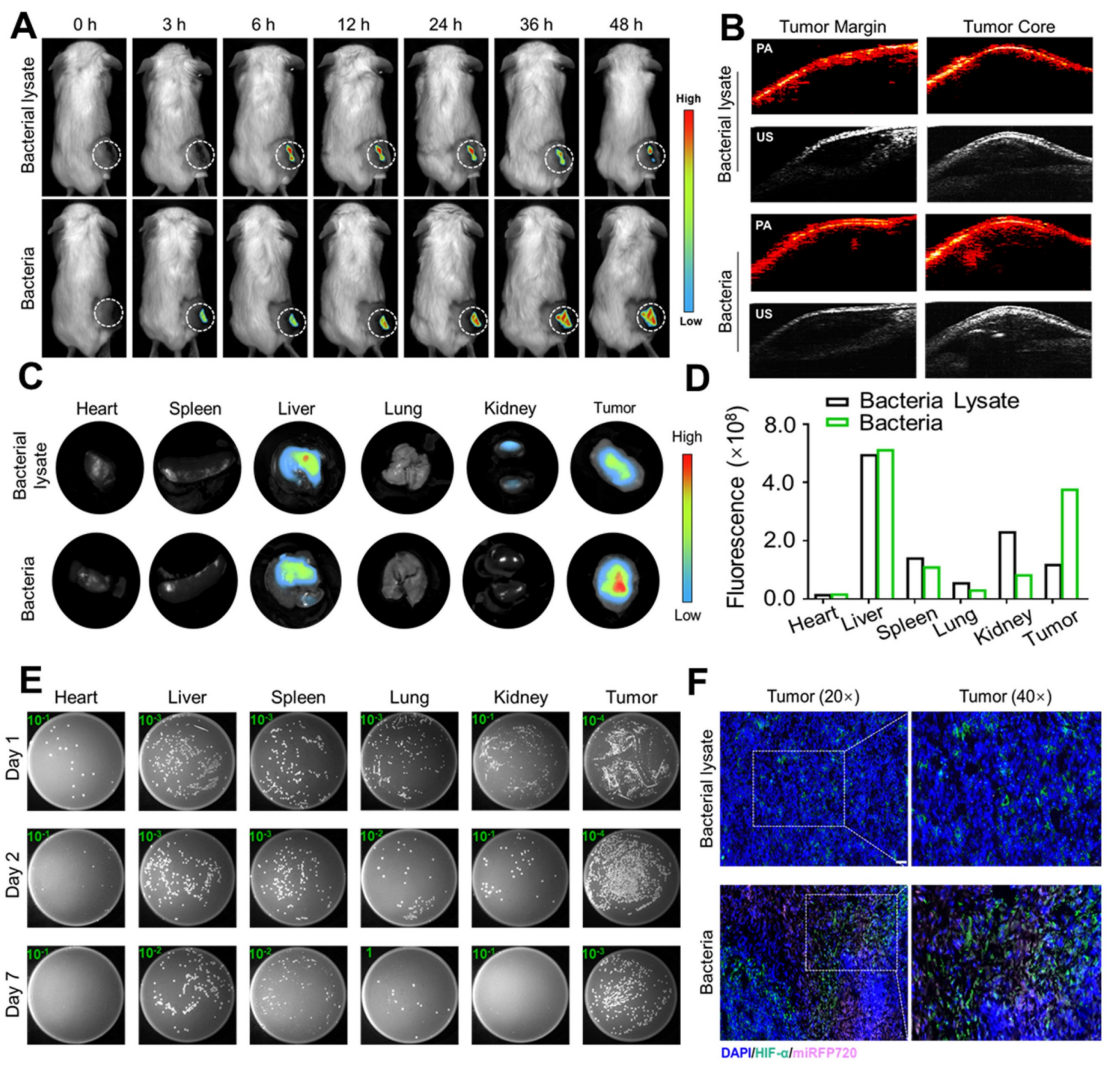
*In vivo* distribution of Cytolysin A-miRFP720 engineered bacteria. (A) The fluorescence image of tumor-bearing mice received bacteria or bacterial lysate (10^8^ CFU/mL per mouse) after intravenous injection within 48 h. (B) The photoacoustic imaging performance of engineered bacteria in the tumor margin and tumor core in both groups 48 h after injection. (C-D) Quantitative analysis of the fluorescence intensity in tumor tissues measured at different time points (0 h, 3 h, 6 h, 12 h, 24 h, 36 h, 48 h) and isolated major organs and tumors after injection of bacteria and bacterial lysate. (E) Homogenates of major organs and tumor tissues were cultivated on LB agar plates at 30 °C after intravenous administration of engineered bacteria at 1 d, 2 d, and 7 d. (F) Fluorescent staining of tumor sections with hypoxia-inducible factor-1α (HIF-1α) (Green), miRFP720 (Pink), and DAPI (Blue). Scale bar=50 μm.

Tumor targeting ability is one of the most attractive advantages of BMTT 21. *Escherichia coli* MG1655 is commonly used in basic research of BMTT due to its advantages of low cost, ease of genetic modification, and low toxicity. Based on the optical characteristics of miRFP720, the *in vivo* biodistribution of Cytolysin A-miRFP720 engineered bacteria on 4T1 tumor-bearing mice received intravenous injection of engineered bacteria was evaluated by the fluorescence signal of miRFP720. First, the engineered bacteria were irradiated by focused ultrasound to express miRFP720, while the same amount of engineered bacteria were lysed to obtain bacterial lysate as the control group. In Figure 5A, the fluorescence signal in the bacteria group was first detected in the tumor area 3 h after administration, whereas in the bacterial lysate group, it was observed at 6 h after administration. Additionally, within 48 h, the fluorescence signal of the bacteria group increased steadily within the tumor, whereas the fluorescence signal of the bacterial lysate group decreased after 24 h. After 48 h of injecting bacteria or bacterial lysate, fluorescence signals were observed in both the liver and tumor of the isolated organs, as shown in Figure 5C. The fluorescence signal in the tumor area of the bacteria group was greater than that of the bacterial lysate group (Figure 5D). Moreover, same trend was also shown in the results of colonies counting of homogenizing tumors and major organs and then culturing the homogenates on plates. Colonies from homogenates of the heart, liver, spleen, lung, and kidney gradually decreased, whereas homogenates of the tumor showed a peak value after 2 days. The number of colonies in the tumor area was higher than that in the main organs. Even on the seventh day after administration, bacteria could still be found in the tumor area (Figure 5E). Fluorescent sections of the tumor (Blue) with hypoxia-inducible factor-1α (HIF-1α, Green) and miRFP720 (Pink) further illustrate the effect of bacterial targeting in the tumor region (Figure 5F), which shown that massive aggregation of bacterial groups occurs in hypoxic regions of tumors.

Photoacoustic imaging is an innovative imaging technology that offers superior deep tissue penetration and imaging accuracy when compared to fluorescence imaging. Due to the photoacoustic imaging effect of miRFP720, photoacoustic tomography was performed to evaluate the distribution of engineered bacteria in tumor tissue. It was demonstrated that the signal in the tumor core was significantly greater compared to that at the tumor margin (Figure 5B). These results indicated that engineered bacteria could target and colonize tumor cores, which had the potential to increase the concentration of therapeutic proteins *in situ*.

**Figure 6:**
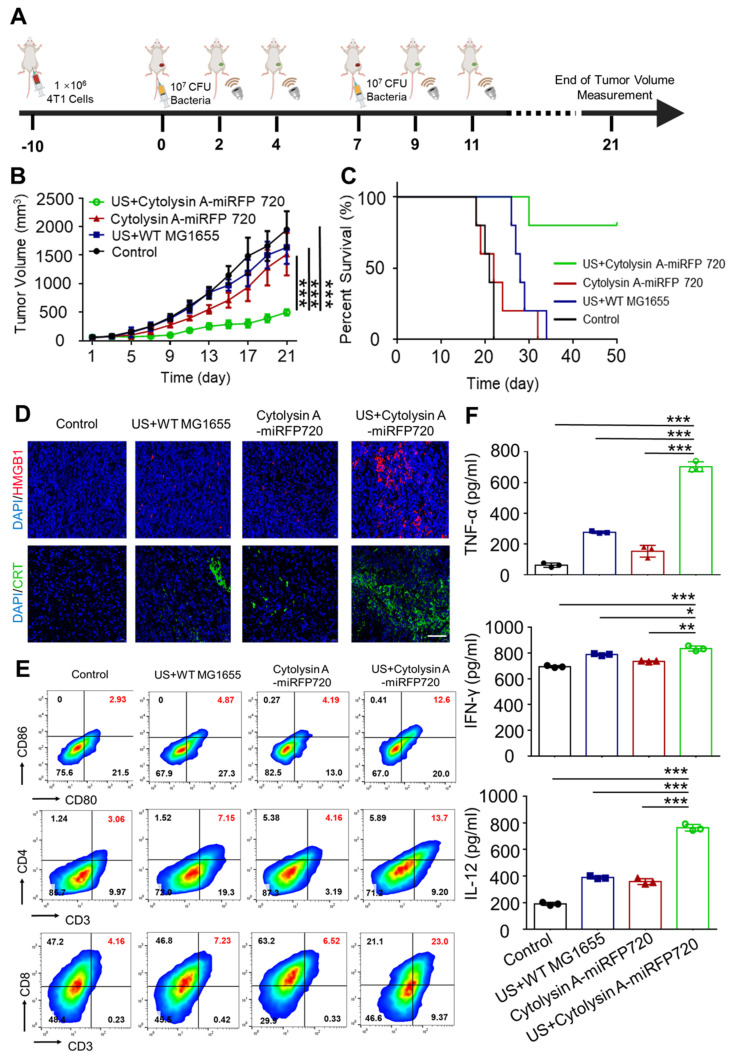
Induction of *in vivo* antitumor immune response by Cytolysin A-miRFP720 engineered bacteria against subcutaneous tumor. (A) Schematic illustration depicting the treatment protocol involving Cytolysin A-miRFP720 engineered bacteria for mice with 4T1 tumors. Ultrasound irradiation was provided on day 2 and 4 after intravenous injection. (B) Tumor volume was measured in groups treated with different therapies (n = 5 per group). (C) Survival rate of tumor-bearing mice was assessed 50 days after treatment with various therapies. (D) Fluorescence images of high mobility group box1 (HMGB1) and calreticulin (CRT) in tumor tissues following varied treatments are shown (Scale bar= 100 μm). (E) Analyze and quantify DCs maturation, as well as CD4+ and CD8a+ T cell populations within tumors using flow cytometry. (F) Quantitative analysis of IFN-γ, TNF-α, and IL-12 levels in tumors of mice receiving different treatments. Data are presented as mean ± SD, and one-way ANOVA with Tukey's test was performed on GraphPad Prism. (^*^p<0.05, ^**^p<0.01, ^***^p<0.001).

To assess the anti-tumor impact of Cytolysin A-miRFP720 engineered bacteria *in vivo*, tumor-bearing mice were divided to four groups were formed among 4T1 tumor-bearing mice: Control with administration of PBS, administration of wild-type MG1655 combined with ultrasound irradiation (US+WT-MG1655), administration of Cytolysin A-miRFP720 engineered bacteria alone and ultrasound irradiation combined with administration of Cytolysin A-miRFP720 engineered bacteria (US + Cytolysin A-miRFP720). All mice were treated with tail vein administration. WT-MG1655 and Cytolysin A-miRFP720 engineered bacteria (10^8^ CFU) were administered on day 1 and 7. At 2 and 4 days after administration, ultrasound irradiation (3.52 MPa, 30 min, 42 °C) of the local tumor site was performed. 21 days after treatment, the tumor volume was significantly reduced in the group treated with US + Cytolysin A-miRFP720 engineered bacteria compared to the other groups, as shown in Figure 6B. Notably, the survival rate of mice treated with US + Cytolysin A-miRFP720 engineered bacteria was greatly improved (Figure 6C). These results indicate that Cytolysin A-miRFP720 engineered bacteria combined with ultrasound can exert ideal anti-tumor effects to inhibit tumor growth. What's more, as the images of H&E slices of the normal organs as well as the counts of WBC and granulocyte, did not change obviously after ultrasound irradiation combined administration of Cytolysin A-miRFP720 engineered bacteria treatment (Figure S3, S4).

Since Cytolysin A was proven to exert ideal immunogenic cell death (ICD) effect *in vitro*, the immune effect of Cytolysin A-miRFP720 engineered bacteria was further evaluated *in vivo*. As figure 6D shown, HMGB1 (Red) and CRT (Green) expression were significantly enhanced in the US + Cytolysin A-miRFP720 engineered bacteria group compared with the other treatment. To elucidate the potential mechanism of the antitumor immune effect of Cytolysin A-miRFP720 engineered bacteria combined with ultrasound, the maturation rate of DCs and the frequency of CD4+ and CD8a+ T cells in the tumors were measured (Figure 6E). The maturation rate of DCs was 2.93%, 4.87%, 4.19%, and 12.6% for the control, US+WT-MG1655, Cytolysin A-miRFP720, and US + Cytolysin A-miRFP720 groups, respectively, indicating that the antigen presentation function was activated. In addition, CD4+ and CD8a+ T cells were both significantly increased in the US + Cytolysin A-miRFP720 group compared to the control, US+WT-MG1655, and Cytolysin A-miRFP720 groups. As for the inflammation cytokine level evaluation, compared with the control group, the expressed levels of IFN-γ, tumor necrosis factor-α (TNF-α), and interleukin-12 (IL-12) in tumor were higher in the US + Cytolysin A-miRFP720 engineered bacteria group (Figure 6F). Collectively, these results suggested that US + Cytolysin A-miRFP720 treatment could effectively activate antitumor immunity, which has the potential to enhance the therapeutic efficiency of immunotherapy.

### *In vivo* antitumor immune response induced by Cytolysin A-miRFP720 engineered bacteria in an orthotopic glioblastoma model

**Figure 7:**
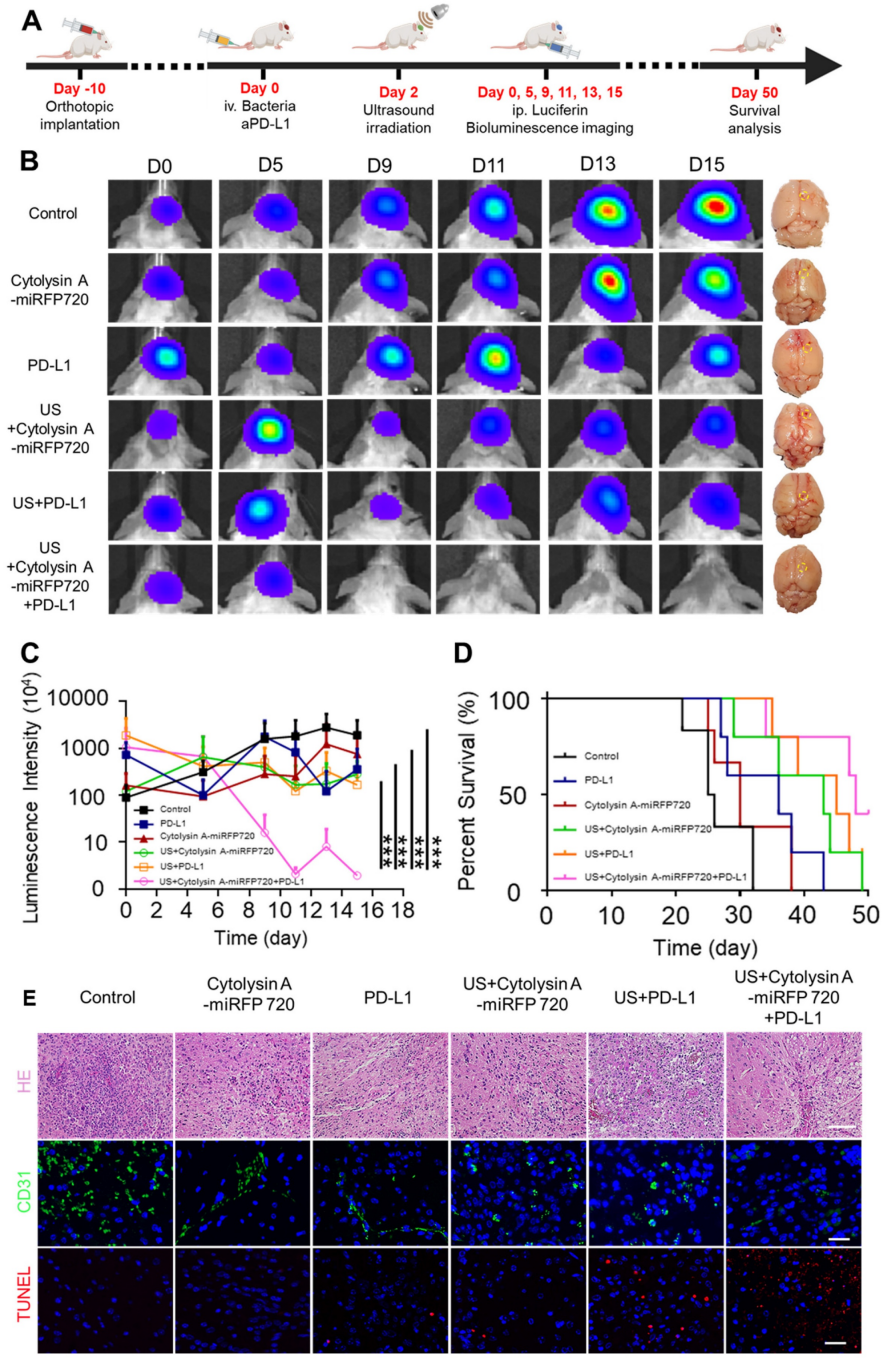
Suppression of orthotopic glioblastoma growth using Cytolysin A-miRFP720 engineered bacteria. (A) Schematic illustration depicting the treatment protocol involving Cytolysin A-miRFP720 engineered bacteria for mice with orthotopic glioblastoma. (B) Luminescence images of tumor-bearing mice and digital images of the brains with tumors isolated at 15 days in the various treatment groups at different time points. (C) Luminescence intensities of tumor-bearing mice within 15 days. (D) Survival curve of all treatment groups. (E) Hematoxylin and eosin (H&E), CD31, and terminal deoxynucleotidyl transferase dUTP nick end labeling (TUNEL) staining of the tumor. Scale bars represent 100 μm, 50 μm, and 50 μm, respectively. Data are presented as mean ± SD, and one-way ANOVA with Tukey's test was performed on GraphPad Prism. (^***^p<0.001).

It has been shown that Cytolysin A-miRFP720 engineered bacteria can activate antitumor immunity. As mentioned, compared to other gene regulation modes, ultrasound has higher tissue penetration and broader applicability for irradiating deep-seated tumors located in the liver, brain, or ovary 22, 23. To evaluate whether ultrasound could successfully induce bacterial expression of therapeutic genes in deep-seated tumors and exhibit optimal therapeutic efficiency, orthotopic glioblastoma-luciferase mice models were selected for further study. Due to the immune response caused by Cytolysin A, as proven before, PD-L1 blockade was also combined, and the therapeutic efficiency of the combined treatment mode was evaluated. Mice with implanted glioblastoma cells expressing luciferase were randomly divided into six treatment groups (n=3 per group): PBS, Cytolysin A-miRFP720 engineered bacteria, aPD-L1, US + Cytolysin A-miRFP720 engineered bacteria, US + aPD-L1, and US + Cytolysin A-miRFP720 engineered bacteria + aPD-L1. Ultrasound irradiation was provided on day 2. The luminescence intensities of mice treated with PBS and bacteria alone increased gradually over 15 days, indicating tumor growth. However, the bioluminescence signal of the aPD-L1, US + Cytolysin A-miRFP720 engineered bacteria, and US + aPD-L1 groups decreased slightly and could still be observed after 15 days (Figure 7B). Notably, the tumor signals gradually decreased by day 9 after treatment in the US + Cytolysin A-miRFP720 engineered bacteria + aPD-L1 group (Figure 7B-C). Within 15 days, the luminescence intensities remained low in this group compared with the other treatment groups, suggesting the highest inhibition of glioblastoma growth with US + Cytolysin A-miRFP720 engineered bacteria + aPD-L1. Compared to aPD-L1 group, the ideal inhibition rate of glioblastoma also indicated that Cytolysin A-miRFP720 engineered bacteria could enhance the efficiency of aPD-L1 immunotherapy. The survival curve showed that the longest survival time was observed in the US + Cytolysin A-miRFP720 engineered bacteria + aPD-L1 group (Figure 7D).

Hematoxylin and eosin (H&E), CD31, and terminal deoxynucleotidyl transferase dUTP nick end labeling (TUNEL) staining were used to study angiogenesis and cell apoptosis in all treatment groups. CD31 staining intensity (green fluorescence) was significantly lower in the US + Cytolysin A-miRFP720 engineered bacteria + aPD-L1 group compared to the other groups (Figure 7E), suggesting positive effects on the ablation of blood vessels in tumor tissues. The stronger TUNEL staining intensity (red fluorescence) observed in the US + Cytolysin A-miRFP720 engineered bacteria + aPD-L1 group represents a stronger apoptotic effect. The results of CD31 and TUNEL staining indicate that the US + Cytolysin A-miRFP720 engineered bacteria + aPD-L1 group had robust effects in preventing angiogenesis and promoting cell apoptosis.

Although the application of ultrasound-controllable image visualization platforms is promising in tumor therapy, there are several problems to be solved. Shapiro *et al.* constructed an optimized ultrasound-responsive state switch, enabling sustained release of a therapeutic payload 24. Enhancing the efficiency of protein expression by modifying components is also a direction that needs to be studied further. In addition, the selection of therapeutic molecules can be further optimized because the cell-killing effect of Cytolysin A therapeutic proteins alone is not sufficient 25. This research demonstrated a positive correlation between therapeutic protein expression and imaging signals, but the imaging signals can only respond to the trend of the therapeutic molecules expressed but not directly quantify the protein levels, which remains to be further studied. Moreover, in this study, optical molecular expression was used to visualize the analysis, but the deep penetration ability of laser is limited. Subsequent studies can consider using other imaging modalities for detection, such as magnetic resonance imaging 26.

## Conclusion

In this study, a real-time imaging guided ultrasound activatable bacteria-mediated tumor therapy mode was established. With the thermal effect of focused ultrasound, Cytolysin A and miRFP720 were concurrently express. Due to the optical imaging property of miRFP720, the therapeutic production Cytolysin A could be monitored *in vivo*. In another words, utilizing optical imaging signal to guide the induction mode of ultrasound could maintain high expression levels of therapeutic molecules in tumor area, which enhances the therapeutic efficiency of tumor as well as aPD-L1 immunotherapy.

## Supplementary Material

Supplementary figures.
