# Supplementary material for: Thermal-responsive ultrasound activatable in situ production of therapeutics for real-time imaging and targeted tumor therapy
Source: Theranostics. 2025 Mar 18;15(9):4212–28. doi: 10.7150/thno.101714 (PMC11980651; doi:10.7150/thno.101714)
Supplement: Supplementary file 1 — Supplementary figures. [file thnov15p4212s1.pdf]

Thermal-responsive ultrasound activatable in situ production of therapeutics for real-time imaging and targeted tumor therapy

Supplementary Information

Meng Du [†][a,b,c,d], Fengyi Zeng [†][a,c,d], Ting Wang [†][a,c,d], Jinghui Fang [e], Yuhao Chen [c,d,f], Likang Hou [a,c,d], Bin Li [a,c,d], Qingrong Xia [a,c,d], Zhen Yuan [f], Fei Yan [g] *, Zhiyi Chen [a,b,c]*

[a] Key Laboratory of Medical Imaging Precision Theranostics and Radiation Protection, College of Hunan Province, The Affiliated Changsha Central Hospital, Hengyang Medical School, University of South China, Changsha, Hunan, China.

[b] Department of Medical Imaging, The Affiliated Changsha Central Hospital, Hengyang Medical School, University of South China, Changsha, Hunan, China.

[c] Institute of Medical Imaging, Hengyang Medical School, University of South China, Hengyang, Hunan, China.

[d] Medical Imaging Centre, The First Affiliated Hospital, Hengyang Medical School, University of South China, Hengyang, Hunan, China.

[e] Third Affiliated Hospital of Guangzhou Medical University, Guangzhou, Guangdong, China.

[f] Faculty of Health Sciences, Centre for Cognitive and Brain Sciences, University of Macau, Taipa, Macau SAR, China.

[g] CAS Key Laboratory of Quantitative Engineering Biology, Shenzhen Institute of Synthetic Biology, Shenzhen Institute of Advanced Technology, Chinese Academy of Sciences, Shenzhen, Guangdong, China.

[†] These authors contributed equally to the work.

[*] Correspondence to: Zhiyi Chen (zhiyi_chen@usc.edu.cn) and Fei Yan (fei.yan@siat.ac.cn)

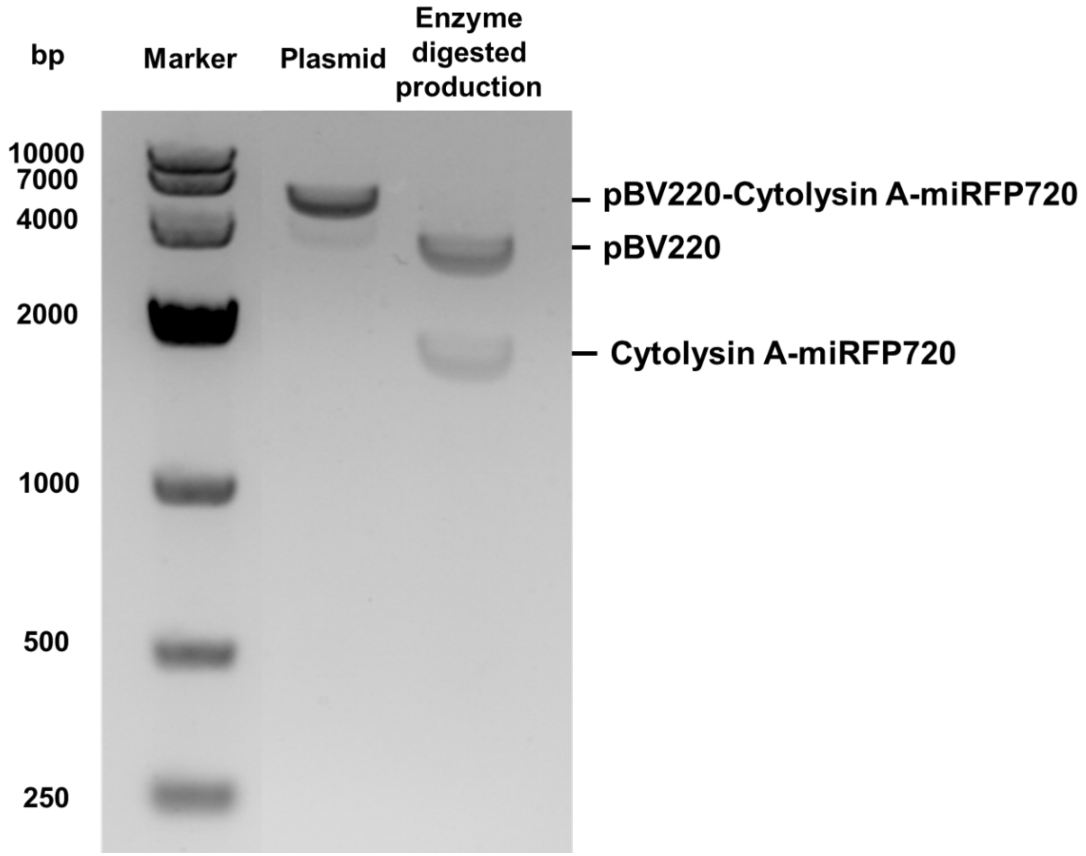

Figure S1. Band distribution of agarose gel electrophoresis of plasmid and plasmid with enzyme digestion.

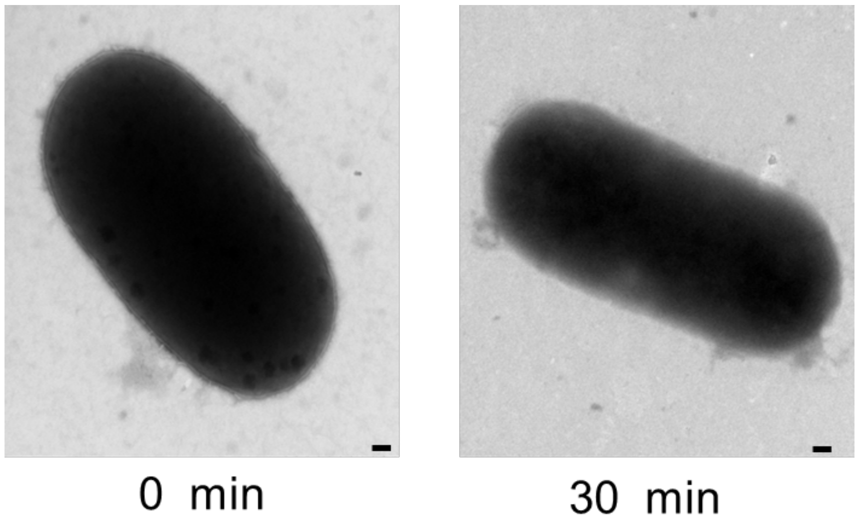

Figure S2. Images of transmission electric microscope of Cytolysin A-miRFP720 bacteria before (0 min) and after ultrasound irradiation (30 min). Scale Bar=500 nm

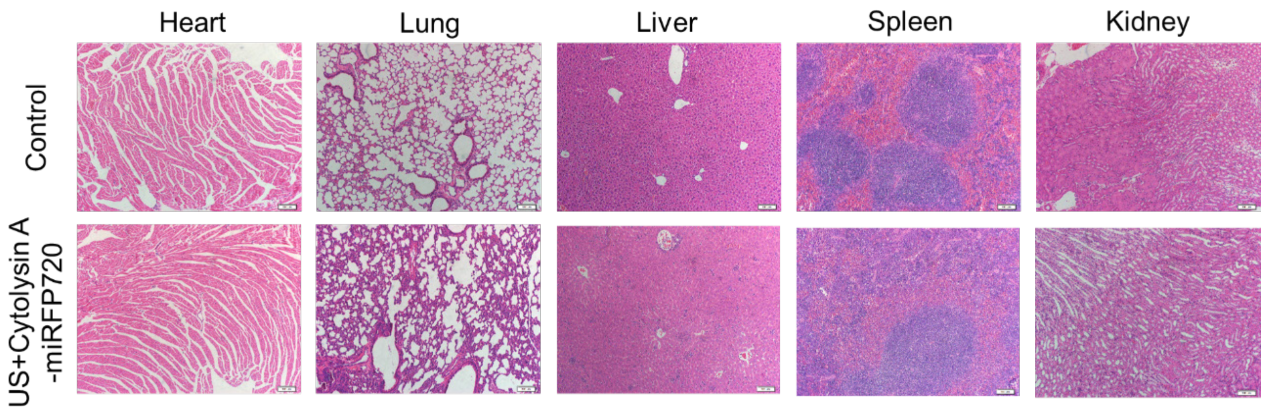

Figure S3. Representative images of H&E staining slices of different treatment. Scale bar=100 μm

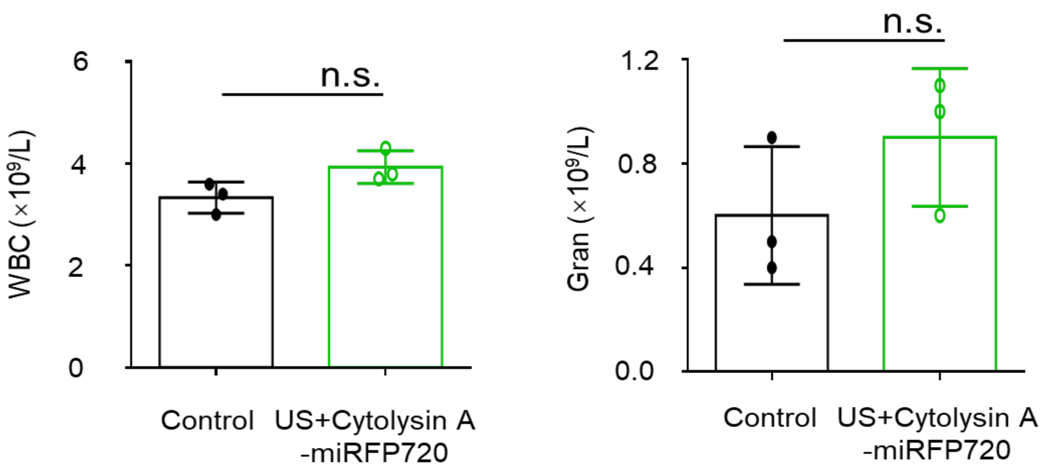

Figure S4. Quantitative analysis of white blood cells and granulocyte after different treatment.
